# Late sodium current in synergism with Ca^2+^/calmodulin-dependent protein kinase II contributes to β-adrenergic activation-induced atrial fibrillation

**DOI:** 10.1098/rstb.2022.0163

**Published:** 2023-06-19

**Authors:** Xiaoyan Liu, Lu Ren, Shandong Yu, Gang Li, Pengkang He, Qiaomei Yang, Xiaohong Wei, Phung N. Thai, Lin Wu, Yong Huo

**Affiliations:** ^1^ Department of Cardiology, Peking University First Hospital, 8, Xishiku Street, West District, Beijing 100034, People's Republic of China; ^2^ Department of Cardiology, Cardiovascular Center, Beijing Friendship Hospital, Capital Medical University, Beijing, 100050, People's Republic of China; ^3^ Division of Cardiovascular Medicine, Department of Internal Medicine, University of California, Davis, CA, 95616, USA; ^4^ Key Laboratory of Medical Electrophysiology of Ministry of Education, Institute of Cardiovascular Research, Southwest Medical University, Luzhou, 646000, People's Republic of China

**Keywords:** atrial fibrillation, β-adrenergic stimulation, late sodium current, Ca^2+^/calmodulin-dependent protein kinase II, eleclazine

## Abstract

Atrial fibrillation (AF) is frequently associated with β-adrenergic stimulation, especially in patients with structural heart diseases. The objective of this study was to determine the synergism of late sodium current (late *I*_Na_) and Ca^2+^/calmodulin-dependent protein kinase (CaMKII)-mediated arrhythmogenic activities in β-adrenergic overactivation-associated AF. Monophasic action potential, conduction properties, protein phosphorylation, ion currents and cellular trigger activities were measured from rabbit-isolated hearts, atrial tissue and atrial myocytes, respectively. Isoproterenol (ISO, 1–15 nM) increased atrial conduction inhomogeneity index, phospho-Na_v_1.5 and phospho-CaMKII protein levels and late *I*_Na_ by 108%, 65%, 135% and 87%, respectively, and induced triggered activities and episodes of AF in all hearts studied (*p* < 0.05). Sea anemone toxin II (ATX-II, 2 nM) was insufficient to induce any atrial arrhythmias, whereas the propensities of AF were greater in hearts treated with a combination of ATX-II and ISO. Ranolazine, eleclazine and KN-93 abolished ISO-induced AF, attenuated the phosphorylation of Na_v_1.5 and CaMKII, and reversed the increase of late *I*_Na_ (*p* < 0.05) in a synergistic mode. Overall, late *I*_Na_ in association with the activation of CaMKII potentiates β-adrenergic stimulation-induced AF and the inhibition of both late *I*_Na_ and CaMKII exerted synergistic anti-arrhythmic effects to suppress atrial arrhythmic activities associated with catecholaminergic activation.

This article is part of the theme issue ‘The heartbeat: its molecular basis and physiological mechanisms’.

## 1. Introduction

Atrial fibrillation (AF) is the most prevalent, progressive tachyarrhythmia and is associated with high morbidity and mortality [1]. Rhythm control strategies for AF include anti-arrhythmic drugs, ablation, surgery and upstream therapy. However, there is still a great need for novel therapeutic drugs owing to suboptimal effects of these therapies, including limited efficacies for all these strategies, serious adverse effects potentially for anti-arrhythmic drugs, and high recurrences rate and potential complications for AF-ablation [2,3]. In patients with heart failure, ischaemic heart disease, or other cardiovascular or chronic renal diseases, the prevalence of AF is high and is more commonly related to the over-activation of the sympathetic nervous system [4]. Excessive activation of the autonomic nervous system is an arrhythmogenic trigger and may be involved in the progression of AF from paroxysmal to persistent [5]. However, β-adrenergic receptor antagonists are insufficient for rhythm control. Inhibition of late sodium current (late *I*_Na_) by ranolazine (RAN) and a more selective inhibitor of late *I*_Na_ eleclazine (ELEC) conferred protection against ischemia- and acetylcholine-induced AF in intact animal models, without significant proarrhythmic effects [6,7].

Late *I*_Na_ was found to be increased in persistent AF and contributed to the pathogenesis of AF in animal models [8,9]. In addition, Ca^2+^/calmodulin-dependent protein kinase (CaMKII)-dependent phosphorylation of Na_V_1.5 resulted in dysregulation of *I*_Na_, i.e. reduced peak *I*_Na_ and enhanced late *I*_Na_, to enhance AF risk was demonstrated in patients with sleep-disordered breathing [10]. A vicious circle of [Ca^2+^]_i_-CaMKII-late *I*_Na_-[Na^+^]_i_ is reported to promote the ventricular and atrial arrhythmias [11,12]. Enhanced CaMKII activity augments late *I*_Na_ by directly phosphorylating Na_v_1.5 [10,13,14]. Theoretically, targeting any part of the circle may break the arrhythmogenic mechanism both in ventricle and atrium [14]. Thus, further evidence of the interaction between late *I*_Na_ and CaMKII on electrical abnormalities and arrhythmogenesis with triggers is important both in research and clinic. The objective of this study was to determine the role of the β activation-CaMKII-late *I*_Na_ pathway in the pathogenesis of AF underlying the β-adrenergic overactivity, and to explore the synergistic effect of combined inhibitions of late *I*_Na_ and CaMKII in alleviation of atrial electrophysiological malfunctions and severity of AF in denervated rabbit hearts and atrial myocytes.

## 2. Methods

### (a) Isolated rabbit heart model

New Zealand White female rabbit (weighing about 2.5–3.5 kg, aged about 3–6 months old) hearts were isolated and perfused in retrograde Langendorff mode with modified Krebs–Henseleit (K–H) solution as previously described [13]. Rabbits were initially anaesthetized with sodium pentobarbitone (50 mg kg^−1^) through the marginal ear vein. After midsternal incision and opening of the pericardium, rabbits were euthanized via exsanguination by excising the hearts for further *ex vivo* study. Modified K–H solution contained (mM): 118 NaCl, 2.8 KCl, 1.2 KH_2_PO_4_, 2.5 CaCl_2_, 0.5 MgSO_4_, 2.0 sodium pyruvate, 5.5 glucose, 0.57 Na_2_EDTA and 25 NaHCO_3_ (adjusted to pH 7.4, bubbled with 95% O_2_ and 5% CO_2_, and warmed to 37°C). Bipolar Teflon-coated electrodes were placed on the epicardium near the sinoatrial node to pace the right atrium at a fixed 4.5 Hz frequency. AF was invoked by programmed stimulation with a 3 ms pulse width and threefold diastolic threshold delivered from a Grass-S88X stimulator (Astro-Med, West Warwick, RI, USA). After initiation of pacing, we had a 10–20 min equilibration period before we began experiments.

### (b) Monophasic action potential and electrocardiogram recording

A pressure-contact Ag-AgCl monophasic action potential (MAP) electrode was placed on the endocardial surface of the left atrial appendage to obtain atrial MAP, and a pseudo 12-lead electrocardiogram (ECG) was recorded using a circular Einthoven-Goldberger ECG electrode system simultaneously (Harvard Apparatus, Inc., Holliston, MA, USA). Electrical signals were digitized in real time by Biopac Wilson MAP and ECG amplifiers (Biopac MP 150, Goleta, CA, USA) [13].

### (c) Electrophysiological experiment protocols

We tested dose-dependent changes in electrophysiological (EP) parameters using various concentrations of isoproterenol (ISO, 1–15 nM) in the same heart. Programmed stimulation (eight S1 stimulations at a cycle length (CL) of 200 ms followed by a premature S2 stimulation at a progressively prolonged CL from the atrial effective refractory period (aERP)) was applied to induce AF. EP measurements include (i) aERP, defined as the shortest S1–S2 interval that resulted in a propagated response after S1 [15]; (ii) AF-related parameters: AF incidence, inducible window (difference between the longest and the shortest S1–S2 interval that successfully invoked AF) and burden (the sum of the duration of inducible AF within the AF window when the pacing CL increased stepwise from the shortest to the longest by 2 ms). Hearts were infused with ISO at a submaximal concentration (15 nM) for 15 min to achieve steady-state EP parameters and induce AF. To test the anti-arrhythmic effects, ISO-infused hearts were treated with RAN and ELEC (MedChem Express, Monmouth Junction, NJ, USA) or KN-93 (Selleckchem, Houston, TX, USA) for 8–15 min at each concentration until a steady-state maximal effect was observed.

### (d) Epicardial activation mapping

Atrial epicardial activation mapping in isolated Langendorff-perfused rabbit hearts was performed by two multi-electrode arrays that contain 32 separated electrodes (arranged in a 4, 6, 6, 6, 6 and 4 grid within an 8 × 8 mm configuration, a 0.1 mm electrode diameter and a 1.6 mm interelectrode distance) connected to a 64-channel amplifier and data acquisition system (EMS64-USB-1002, MappingLab Inc., City of Industry, CA, USA). The inhomogeneity index (IHI) was calculated at the fixed rate pacing and at the first beat of AF [16]. Isochrones were derived by an off-line analysis program (EMapScope 3.0, MappingLab Inc., City of Industry, CA, USA). Data were sampled at 10 kHz per channel.

### (e) Immunoprecipitation and Western blot analysis

Left atrial tissue was homogenized using a tissue lyser. Levels of phospho-CaMKII at T286 (no. 12716S, Cell Signaling Technology, Danvers, MA, USA) and human cardiac voltage-gated sodium channel (no. sc-271255-1, NaV1.5, Santa Cruz Biotechnology, Santa Cruz, CA, USA) were determined by immunoprecipitation and Western blot [13]. In detail, non-specific proteins were removed by mixing the protein sample and IgG of the same species used in the subsequent immunoprecipitation reaction and Protein G Plus/Protein A-Agarose (no. IP-10-10mlCN, Millipore, MA, USA). Then tissue lysates were incubated with magnetic beads covalently conjugated with anti-Na_v_1.5 Ab and anti-phospho-protein kinase A (PKA) substrate Ab (RRXS*/T*, no. 9624, Cell Signaling Technology) at 4°C overnight. Sample buffer mixed with the washed beads was heated at 95°C for 5 min and subjected to sodium dodecyl sulfate-polyacrylamide gel electrophoresis. The proteins were blotted with rabbit anti-Na_v_1.5 Ab and anti-phospho-PKA substrate Ab, respectively. The relative intensity of individual bands was visualized using an electrochemiluminescence detection system (Millipore, Darmstadt, Hessen, Germany) and quantified using ImageJ software. The ratio for the control was assigned a value of 1.

### (f) Whole-cell patch-clamp measurements

Atrial myocytes were enzymatically dissociated. Whole-cell voltage and current patch-clamps (EPC-10, Heka Electronic, Lambrecht, Pfalz, Germany) were used to measure late *I*_Na_ and action potentials (APs) as previously described (filtered at 1 kHz, digitized at 10 kHz) [13]. All experiments were conducted at room temperature. We began data acquisition after 5 min of drug exposure.

For late *I*_Na_ recording, bath solution contained (in mM) 135 NaCl, 5.4 CsCl_2_, 1.8 CaCl_2_, 1 MgCl_2_, 0.3 BaCl_2_, 0.33 NaH_2_PO_4_, 10 HEPES, 10 glucose and 0.001 nicardipine (adjusted to pH 7.2 with CsOH). The pipette solution contained (in mM) 120 CsCl_2_, 1 CaCl_2_, 5 MgCl_2_, 5 Na_2_ATP, 10 TEA-Cl, 10 EGTA and 10 HEPES (adjusted to pH 7.3 with CsOH). Late *I*_Na_ was recorded by a 300 ms depolarizing pulse to −20 mV from a holding potential of −90 mV, and measured at 200 ms after initiation of the depolarization step. Measured currents were normalized to the membrane capacitance.

For AP recordings, bath solution contained (mM) 144 NaCl, 5.6 KCl, 1.2 MgCl_2_, 1.8 CaCl_2_, 5 HEPES and 11 glucose (adjusted to pH 7.4 with NaOH), and the pipettes were filled with (in mM) 5 NaCl, 30 KCl, 10 K-aspartate, 5 creatine phosphate, 10 HEPES, 10 EGTA, 0.05 cAMP and 5 Mg-ATP (adjusted to pH 7.2 with KOH). APs were continuously elicited by square current pulses of 200–300 pA amplitude and 10 ms duration at a stimulation frequency of 1 Hz. The AP durations (APD) and incidence of early afterdepolarizations (EADs) and delay afterdepolarizations (DADs) were measured.

### (g) Statistical analysis

All statistical analyses were performed using GraphPad Prism (version 6.0, GraphPad Software, Inc., San Diego, CA) and SPSS (version 19.0, SPSS Inc., Chicago, IL). The normality of the continuous variable distribution was evaluated using the Shapiro–Wilk test. Continuous data were expressed as the mean ± s.e.m. and non-normally distributed data were expressed as median with interquartile range. Normally distributed values were analysed using Student's *t*-tests or one- or two-way repeated measures ANOVA, followed by the Student–Newman–Keuls test, while non-normally distributed values were compared using Mann–Whitney tests. Categorical variables were summarized by absolute numbers or percentages and compared with *χ*^2^ tests. A two-sided *p* < 0.05 was considered statistically significant.

## 3. Results

### (a) Propensity of isoproterenol-induced atrial fibrillation was exacerbated in sea anemone toxin II pretreated hearts

**Figure 1.**
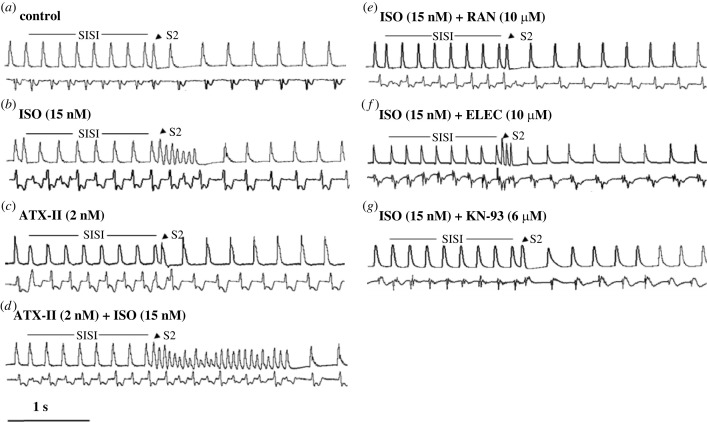
Representative records of AF in the absence (control) and presence of isoproterenol (ISO; 15nM) treated with either ranolazine (RAN), eleclazine (ELEC), or KN-93 in rabbit-isolated hearts. Hearts were paced at 4.5 Hz in right atrium. MAP (upper recordings in each panel) and an ECG (lower recordings in each panel) were recorded simultaneously. The MAP and ECG traces under programmed stimulation of hearts treated with either vehicle or drugs as shown on the top of each panel.

**Figure 2.**
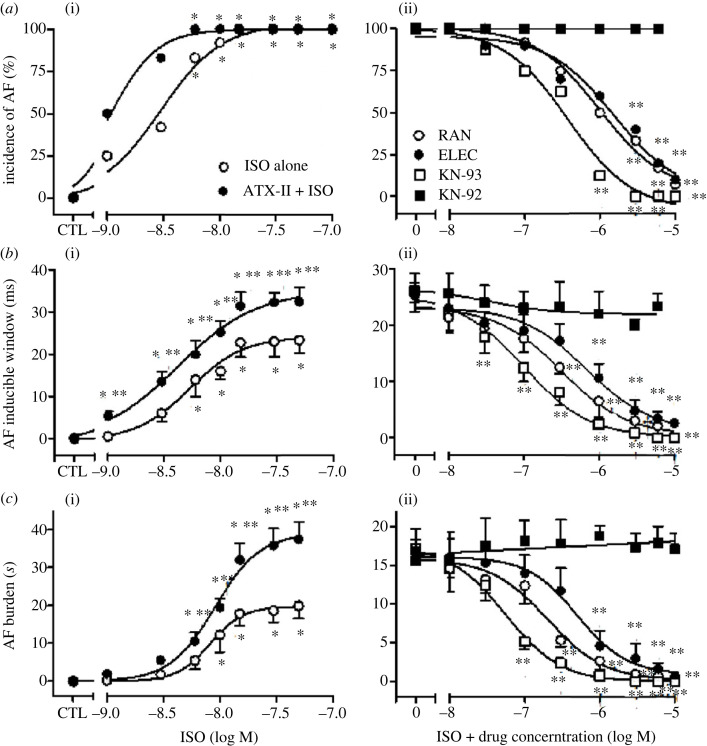
Concentration–response relationships of drugs on AF incidence (*a*), inducible window (*b*) and burden (*c*) induced by isoproterenol (ISO) in the absence (*n* = 12) and presence of sea anemone toxin II (ATX-II; 2 nM, *n* = 6) and treated with either ranolazine (RAN; *n* = 8), eleclazine (ELEC; *n* = 8), KN-93 (*n* = 8), or KN-92 (*n* = 6) in rabbit-isolated hearts. (i) Concentration–response relationships of ISO on AF in the absence and presence of ATX-II (2 nM); (ii) concentration–response relationships of RAN, ELEC, KN −93 or KN-92 on AF induced by ISO (15 nM). 0, ISO alone; *p < 0.05 versus control (CTL); **p < 0.05 versus ISO alone.

As expected, we did not observe AF when programmed atrial stimulation was administered under control conditions (figure 1*a*). Relative to baseline, perfusion with increasing concentrations of ISO (1–15 nM) decreased aERP (from 79.4 ± 2.1 to 52.0 ± 3.8 ms, *p* < 0.05, not shown), increased the incidence, inducible window, and burden of stimulation-induced AF, in a dose-dependent manner (*n* = 12, *p* < 0.05; figure 1*b* and figure 2*a–c*(i)). To test the effect of enhanced late *I_Na_* on ISO-induced AF, we perfused hearts with ISO in the presence of sea anemone toxin II (ATX-II). Pretreatment with ATX-II (2 nM), which was insufficient to induce any atrial arrhythmias, prolonged aERP from 79.4 ± 3.1 to 84.7 ± 4.0 ms (*p* < 0.05; electronic supplementary material, figure S1A) and accentuated the propensities of ISO-induced AF (*n* = 6, *p* < 0.05; figure 1*c,d* and figure 2*a–c*(i)). The incidence of AF was increased to 100% with 15 nM ISO alone, and with a lower concentration of ISO (6 nM) in the presence of ATX-II (figure 2*a*(i)). As the concentration of ISO increases, curves of AF inducible window and burden shifted upwards and leftwards with application of 2 nM ATX-II. The AF inducible window and burden were increased by 7.2 ± 1.5 ms and 14.4 ± 2.1 s, respectively, relative to hearts treated with 15 nM ISO alone (*p* < 0.05; figure 2*b,c*(i)). Moreover, the EC_50_ values for ISO to induce AF incidence, inducible window and burden were significantly lower in the presence than in the absence of ATX-II (1.1 versus 3.0, 4.2 versus 5.3, and 8.2 versus 9.0 nM, respectively, *n* = 6, *p* < 0.05). Taken together, these data show that enhancement of late *I_Na_* accentuated ISO-induced AF.

**Figure 3.**
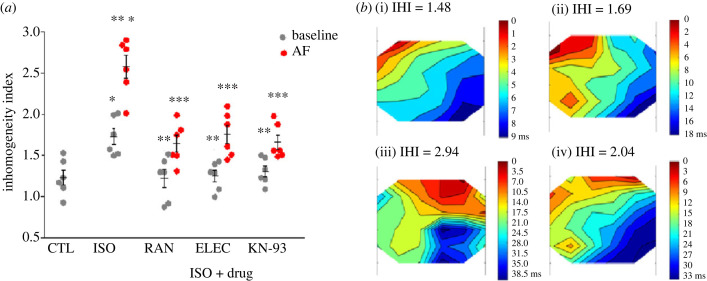
Effects of drugs on inhomogeneity of atrial conduction. (*a*) The index of inhomogeneity (IHI) at a fixed rate (baseline) and at the first beat of AF in hearts treated with either isoproterenol (ISO; 15 nM) alone or ISO + ranolazine (RAN; 10 µM, *n* = 6), ISO + eleclazine (ELEC; 10 µM, *n* = 6) or ISO + KN-93 (3 µM, *n* = 6). (*b*) Representative examples of epicardial conduction properties of left atrium by multi-electrode array in the absence (i) and presence (ii) of ISO at baseline, and treated with ISO (iii) and ISO + ELEC (10 µM) (iv) at the first beat of AF. *p < 0.05 versus control (CTL); **p < 0.05 versus ISO at baseline; ***p < 0.05 versus ISO + drugs at the first beat of AF.

Infusion with 15 nM ISO increased the IHI from 1.2 ± 0.03 to 1.7 ± 0.03 at constant pacing rate (4.5 Hz, *n* = 6, *p* < 0.05, figure 3*a*). Compared with an orderly spread of excitation beginning with localized stimulation-induced activation, the excitation pattern at the first beat of AF resulted in an inhomogeneous mode, with the IHI increased by 108% (from 1.2 ± 0.03 to 2.6 ± 0.05 ms) (*n* = 6, *p* < 0.05, figure 3).

### (b) Inhibition of late sodium current by ranolazine and eleclazine suppressed isoproterenol-induced atrial fibrillation

Late *I*_Na_ inhibitors RAN and ELEC were used to evaluate the effects on ISO-induced AF. RAN significantly inhibited the incidence, inducible window, and burden of ISO-induced AF, with IC_50_ values of 1.0, 0.3 and 0.2 µM, respectively (*n* = 8; figure 2*a–c*(ii)). Similar anti-arrhythmic effects were also observed for ELEC, with IC_50_ values of 1.5, 0.7 and 0.5 µM, respectively (*n* = 8; figure 2*a–c*(ii)). At a concentration of 10 µM, both RAN and ELEC abolished AF in all hearts studied (figure 1*e,f*), suggesting that late *I*_Na_ may participate in ISO-induced AF. Compared with ISO alone, the prolongation of aERP by 10 µM RAN and ELEC were 29.8 ± 2.6 and 16.9 ± 2.4 ms, respectively (*p* < 0.05; electronic supplementary material, figure S1B).

Furthermore, both RAN and ELEC at 10 µM decreased IHI from 1.7 ± 0.03 to 1.2 ± 0.04 and 1.3 ± 0.02 at fixed rate pacing and from 2.6 ± 0.05 to 1.6 ± 0.04 and 1.7 ± 0.04 at the first beat of AF, respectively (*n* = 6, *p* < 0.05; figure 3).

### (c) Inhibition of Ca^2^^+^/calmodulin-dependent protein kinase by KN-93 abolished isoproterenol-induced atrial fibrillation

In hearts treated with ISO (15 nM), KN-93 (0.01–10 µM) decreased the incidence, inducible window and burden of AF in a concentration-dependent manner, with IC_50_ values of 0.4, 0.1 and 0.06 µM, respectively (*n* = 8; figure 2*a–c*(ii)). KN-93 at 6 µM abolished AF in all hearts studied (figure 1*g*). KN-93 at 10 µM also substantially prolonged aERP by 28.3 ± 2.1 ms in the presence of ISO (*p* < 0.05; electronic supplementary material, figure S1B). By contrast, inactive analogue KN-92 had no effect on these parameters (*n* = 6; figure 2*a–c*(ii)). Moreover, with the perfusion of 3 µM KN-93 in the presence of ISO, IHI decreased by 0.4 ± 0.02 at fixed rate pacing and 1.0 ± 0.03 at the first beat of AF, respectively (*n* = 6, *p* < 0.05; figure 3).

### (d) Attenuation of the isoproterenol-enhanced activity of Na_v_1.5 and Ca^2^^+^/calmodulin-dependent protein kinase in atria by ranolazine, eleclazine and KN-93

**Figure 4.**
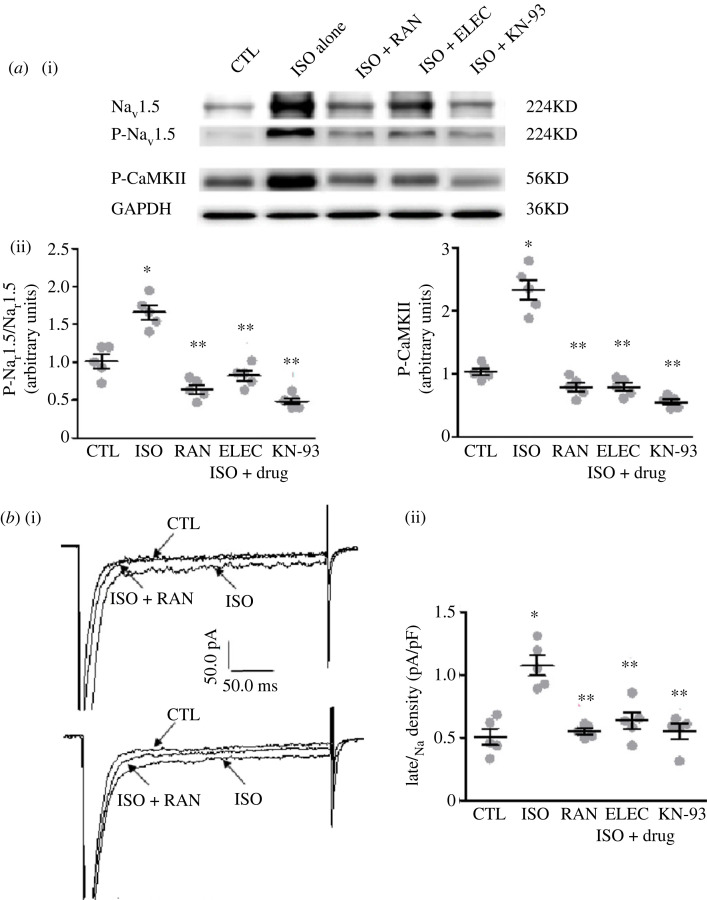
Reverse effects of late *I*_Na_ or CaMKII inhibitors on phosphorylation of Na_v_1.5 and CaMKII (*a*) and late *I*_Na_ (*b*) treated with vehicle (CTL), isoproterenol (ISO; 15 nM) or ISO + ranolazine (RAN), eleclazine (ELEC) and KN-93 (3 µM) respectively. (*a*) Representative Western blots (i) and the relative levels (ii) phospho-Na_V_1.5 (P-Na_v_1.5; *n* = 5) and phospho-CaMKII (P-CaMKII-δ; *n* = 5, normalized to GAPDH and expressed relative to normal levels) protein expression are presented. (*b*) Representative records (i, *n* = 5) and summarized absolute values (ii, *n* = 5) of late *I*_Na_ are presented. *p < 0.05 versus control (CTL); **p < 0.05 versus ISO alone.

ISO dramatically increased phospho-Na_V_1.5 at serine 573 and threonine 17 loci and phospho-CaMKII protein expression levels in atria by 65% and 135%, respectively (*n* = 5, *p* < 0.05; figure 4*a*). By contrast, RAN (10 µM), ELEC (10 µM) and KN-93 (6 µM) attenuated these effects, reducing phospho-Na_V_1.5 by 62%, 48% and 70%, respectively, and of phospho-CaMKII by 67%, 66% and 75%, respectively (*p* < 0.05; figure 4*a*). Representative protein bands expression in each group are displayed in figure 4*a*(i).

### (e) Is oproterenol-induced augmentation of late sodium current and trigger activities were attenuated by either late sodium current or Ca^2+^/calmodulin-dependent protein kinase inhibitors

ISO (15 nM) significantly increased late *I*_Na_ in isolated atrial myocytes by 87%, from an amplitude of 0.57 ± 0.01 to 1.04 ± 0.02 pA pF^−1^ (*n* = 5, *p* < 0.05; figure 4*b*). The increases in late *I*_Na_ induced by the continued presence of ISO were reversed by RAN (10 µM), ELEC (10 µM) or KN-93 (3 µM) by 49%, 41% and 48%, respectively (*n* = 5, *p* < 0.05; figure 4*b*).

**Figure 5.**
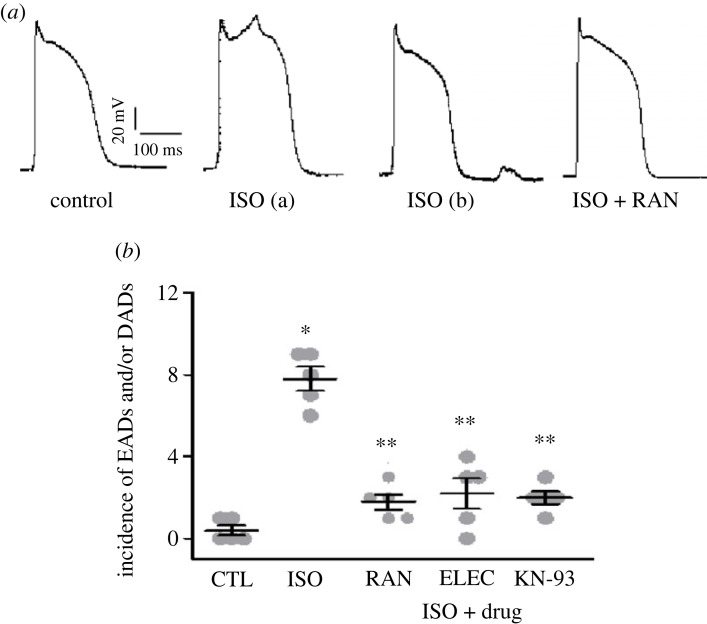
Late *I*_Na_ and CaMKII inhibitors reduced early/delay afterdepolarizations (EADs/DADs) in atrial myocytes induced by isoproterenol (ISO; 15 nM). Original records of action potential ((*a*): EAD (a), DAD (b), *n* = 10) and summarized data ((*b*), *n* = 5) are presented. *p < 0.05 versus control (CTL); **p < 0.05 versus ISO alone.

There were no substantial changes in APD after ISO infusion in atrial myocytes (201.7 ± 24.6 ms versus 213.3 ± 30.1 ms, *n* = 10, *p* > 0.05), as well as the AP morphology (figure 5*a*). ISO elicited EADs/DADs with an incidence of 7.8 ± 0.2 in 5 min (*n* = 5, *p* < 0.05; figure 5). Co-treatment with RAN (10 µM), ELEC (10 µM) or KN-93 (3 µM) suppressed almost all cellular trigger activities (*n* = 5, *p* < 0.05; figure 5*b*).

### (f) The combined effect of late sodium current and Ca^2+^/calmodulin-dependent protein kinase inhibition on isoproterenol-induced atrial fibrillation

**Figure 6.**
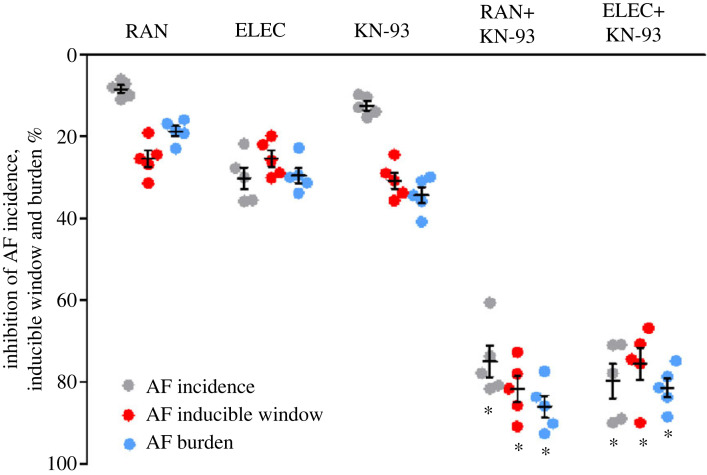
The combined used of late *I*_Na_ and CaMKII inhibitors had synergistic inhibitive effects on isoproterenol (ISO)-induced AF. Combined use of KN-93 (0.03 µM) with either ranolazine (RAN; 0.1 µM) or eleclazine (ELEC; 0.3 µM) were superior to that when each drug was used alone or the sum of the effects of two drugs (either KN-93 and RAN or KN-93 and ELEC, *n* = 5) in reducing the incidence, inducible window and burden of AF in the continued presence of ISO. *p < 0.05 versus individual effects or the sum (not shown) of the effects of two agents when they were used individually.

When used alone, 0.1 µM RAN, 0.3 µM ELEC or 0.03 µM KN-93 attenuated the ISO-induced AF incidence, inducible window and burden by 8–30% (figure 6). The combination of KN-93 and RAN inhibited the ISO-induced increase in AF incidence, inducible window and burden by 75%, 81.7% and 85.9%, respectively (*n* = 5, *p* < 0.05 versus individual effects or their sum; figure 6). A similar result was obtained in hearts treated with the combination of KN-93 and ELEC (*n* = 5, *p* < 0.05 versus individual effects or their sum; figure 6). Consistent with the electrophysiological data, the combination of KN-93 and either RAN or ELEC at the mentioned concentrations generated greater inhibitory effects on phospho-Na_V_1.5 and phospho-CaMKII protein expression levels and late *I*_Na_ than each inhibitor alone or their sum (*n* = 5; electronic supplementary material, figure S2), suggesting that the inhibitors of late *I*_Na_ and CaMKII synergistically act to inhibit ISO-induced AF.

## 4. Discussion

The main findings of this study are (i) β-adrenergic activation caused AF and abnormal atrial electrical activity, and late *I*_Na_ enhancer ATX-II promoted proarrhythmic activities of ISO in atria; (ii) the underlying mechanisms of ISO-induced AF may be attributed, at least in part, to augmentation of late *I*_Na_ by phosphorylation of Na_V_1.5 and CaMKII-δ, and (iii) low concentrations of a late *I_Na_* inhibitor (either RAN or ELEC) with a CaMKII inhibitor, KN-93, synergistically exerted anti-arrhythmic effects.

In Langendorff-perfused animal hearts [17] and patients with paroxysmal AF [18], AF can be induced more often via programmed stimulation after administration of ISO. The proarrhythmic effects of ISO are principally mediated by activation of a β-adrenergic receptor, resulting in increased *I*_Ca,L_ and elevation of [Ca^2+^]_i_ [19]. ISO can also activate CaMKII and PKA to enhance late *I*_Na_ through proteins activated by cAMP-dependent pathways directly and through increasing [Ca^2+^]_i_ indirectly [20]. The positive feedback circle of [Ca^2+^]_i_-CaMKII-late *I*_Na_-[Na^+^]_i_ could progressively facilitate the genesis of DADs via activation of a sodium-calcium exchanger, leading to transient inward currents (*I*_ti_) and EADs via enhancement of *I*_Ca,L_ [2,21], thus promoting atrial hypertrophy, dilatation and AF progression [22]. In addition, ISO shortened aERP in Langendorff-perfused rat hearts, thereby increasing the availability of reactivation, the inhomogeneity of conduction and the origination of re-entry circuits [23]. Taken together, in line with our data, the underlying arrhythmogenic mechanisms of ISO are involved in focal ectopic activity and reentry, and CaMKII plays a crucial role in the initiation of β-adrenergic stimulation-induced AF and might also contribute to the maintenance of AF [24].

### (a) Late sodium current and atrial fibrillation

Both endogenous and enhanced late *I*_Na_ has been suggested as an important modulator of arrhythmias in patients with a variety of conditions [25]. We found that ISO significantly augmented late *I*_Na_ by twofold and promoted AF. These effects were attenuated by late *I*_Na_ inhibitors. Thus, the enhancement of late *I*_Na_ contributed to the arrhythmogenesis induced by ISO. ATX-II (2 nM) increased the baseline level of late *I*_Na_ and thereby reduced repolarization reserve but was insufficient to induce AF. Results in the present study are in agreement with the findings of previously reported studies [13,26,27] and extend these results by revealing that ATX-II pretreatment accentuated the propensities of ISO-induced AF to a greater extent than that observed with ISO alone. These results indicate that the integrative effect of ATX-II and ISO on late *I*_Na_ caused AF, suggesting that enhancement of late *I*_Na_ was a potential proarrhythmic factor. When the repolarization reserve was reduced (e.g. increases in late *I*_Na_), ISO increased the occurrence of arrhythmias.

Increased late *I*_Na_ potentially promotes arrhythmogenesis via both triggered and re-entrant mechanisms [28]. The increases of inhomogeneity of conduction, abnormal cellular trigger activities and AF caused by ISO were attenuated or completely abolished by RAN and ELEC at concentrations that late *I*_Na_ was selectively inhibited. The incomplete reversal by agents that inhibit late *I*_Na_ may be owing to the involvement of other ion currents (e.g. peak *I*_Na_, *I*_Kr_ and *I*_K1_ and L-type calcium channels were affected by ISO directly) in hearts treated with ISO [29].

### (b) Ca^2+^/calmodulin-dependent protein kinase and late sodium current

The proarrhythmic mechanism underlying AF entails increased late *I*_Na_, which probably contributes to triggered AF by increasing [Ca^2+^]_i_ secondary to the increase in intracellular sodium concentration [30], especially in some animal models of AF and in patients with chronic AF [8,31]. The interaction between late *I_Na_* and CaMKII has been demonstrated in calcium-related ventricular arrhythmias [13] and ATX-II-induced AF [27].

In this study, phospho-Na_V_1.5 and phospho-CaMKII protein expression levels were increased by ISO. CaMKII overactivity increased late *I*_Na_ by phosphorylating Na_v_1.5 in rabbit ventricular myocytes [13]. On the other hand, increased late *I*_Na_ disrupts intracellular Ca^2+^ handling [14] which consequently increases the activity of CaMKII [27,28]. Furthermore, an increase in late *I*_Na_ has been shown to elevate intracellular Na^+^ and Ca^2+^, which can be attenuated with late *I*_Na_ inhibitors [32]. Consistent with these previous findings, our study demonstrated that increased levels of the phospho-Na_V_1.5 and phospho-CaMKII proteins were significantly reduced by late *I*_Na_ inhibitors. Our results suggest that the activation of CaMKII induced by ISO resulted in the increase in late *I*_Na_, and consequently promoted the proarrhythmic effect of CaMKII in hearts perfused by ISO.

### (c) Therapeutic potential with inhibition of both Ca^2+^/calmodulin-dependent protein kinase and late sodium current

Effective treatment of AF remains a challenge in this field. Increasing evidence has shown that late *I*_Na_ inhibition may be a promising, anti-arrhythmogenic, alternative strategy to improve AF with CaMKII hyperactivation [33]. A promiscuous nature with a large number of off-target effects on ion channels and β-blocker activity may limit anti-arrhythmic potential of RAN in atria [34]. ELEC, with minimal effects on other cardiac ion channels, confers protection against ischemia-induced AF during adrenergic stimulation without negative inotropic effects [6].

Previous studies have indicated the existence of a proarrhythmic, synergistic relationship between late *I*_Na_ and CaMKII [12]. In the present study, RAN or ELEC and KN-93 at low concentrations alone modestly inhibited ISO-induced AF in isolated hearts. The key finding was that a combination of KN-93 with either ELEC or RAN is superior to the individual effects of each inhibitor on the suppression of arrhythmic activities, which also suggests an interaction between ISO-enhanced CaMKII and late *I*_Na_. Enhancement of late *I*_Na_ by ATX-II to induce AF was previously reported by Liang *et al.* in rat atrial preparations [27]. The results in this study confirmed that β-adrenergic activations to induce AF were associated with a synergistic effect between increased late *I*_Na_ and the activation of CaMKII with possible underlying mechanisms of a vicious circle of the β-receptor activation-CaMKII-late *I*_Na_ pathway. Activation of CaMKII lead to the phosphorylation of Na_v_1.5 and enhancement of late *I*_Na_, which forms the positive feedback cycle between activated CaMKII and enhanced late *I*_Na_ with the presence of a relatively low concentration of ATX-II to induce AF. CaMKII lead to the phosphorylation of Na_v_1.5 and enhancement of late *I*_Na_, which forms the positive feedback cycle between activated CaMKII and enhanced late *I*_Na_ to induce AF. Additionally, β-adrenergic stimulation may augment late *I*_Na_ via other mechanisms independent of CaMKII [29]. In this scheme, both CaMKII and late *I*_Na_ were activated by β-adrenergic stimulation, and augmented late *I*_Na_ was also mediated by activation of CaMKII, providing a mechanism for the combined effect of inhibitors of CaMKII and late *I*_Na_ on AF prevention. Using a combination of CaMKII and late *I*_Na_ inhibitors at lower dosages to achieve the same outcomes as those provided by mono-therapy can potentially reduce the risk of unexpected drug side effects. This finding may provide a new therapeutic strategy for β-adrenergic stimulation-related AF.

## 5. Limitations

The *ex vivo* effects of ISO may be different from the activation of the sympathetic nervous system in AF *in vivo.* ISO may cause phosphorylation and/or activation of multiple downstream targets which were not investigated individually. Abnormal intracellular calcium and sodium concentrations caused by either ISO or late *I*_Na_ enhancement were not measured in this study.

## 6. Conclusion

Enhancement of late *I_Na_* represents an important factor in mediating β-adrenergic stimulation-mediated AF, which may be associated with the phosphorylation of CaMKII and Na_V_1.5. Inhibitions of CaMKII-late *I*_Na_ are effective in synergistic mode in suppressing AF associated with catecholaminergic activation.

## Data Availability

The data are available from the Dryad Digital Repository: https://doi.org/10.5061/dryad.1ns1rn8xn [35]. Data are also provided in the electronic supplementary material [36].
